# Sunscreens Containing Cyclodextrin Inclusion Complexes for Enhanced Efficiency: A Strategy for Skin Cancer Prevention

**DOI:** 10.3390/molecules26061698

**Published:** 2021-03-18

**Authors:** Layan Dahabra, Grace Broadberry, Adam Le Gresley, Mohammad Najlah, Mouhamad Khoder

**Affiliations:** 1 School of Life Sciences, Pharmacy and Chemistry, SEC Faculty, Kingston University, Kingston-upon-Thames KT1 2EE, UK; layan.dahabra@gmail.com (L.D.); k1524065@kingston.ac.uk (G.B.); a.legresley@kingston.ac.uk (A.L.G.); 2 Pharmaceutical Research Group, School of Allied Health, Faculty of Health, Education, Medicine and Social Care, Anglia Ruskin University, Bishops Hall Lane, Chelmsford CM1 1SQ, UK; mohammad.najlah@aru.ac.uk

**Keywords:** natural antioxidants, UV filters, cyclodextrins, hydroxypropyl-β-cyclodextrin, inclusion complex, sunscreens, quercetin, trans-resveratrol

## Abstract

Unprotected exposure of skin to solar ultraviolet radiation (UVR) may damage the DNA of skin cells and can lead to skin cancer. Sunscreens are topical formulations used to protect skin against UVR. The active ingredients of sunscreens are UV filters that absorb, scatter, and/or reflect UVR. Preventing the formation of free radicals and repairing DNA damages, natural antioxidants are also added to sunscreens as a second fold of protection against UVR. Antioxidants can help stabilise these formulations during the manufacturing process and upon application on skin. However, UV filters and antioxidants are both susceptible to degradation upon exposure to sunlight and oxygen. Additionally, due to their poor water solubility, natural antioxidants are challenging to formulate and exhibit limited penetration and bioavailability in the site of action (i.e., deeper skin layers). Cyclodextrins (CDs) are cyclic oligosaccharides that are capable of forming inclusion complexes with poorly soluble drugs, such as antioxidants. In this review, we discuss the use of CDs inclusion complexes to enhance the aqueous solubility of antioxidants and chemical UV filters and provide a protective shield against degradative factors. The role of CDs in providing a controlled drug release profile from sunscreens is also discussed. Finally, incorporating CDs inclusion complexes into sunscreens has the potential to increase their efficiency and hence improve their skin cancer prevention.

## 1. Introduction

Ultraviolet radiation (UVR), mainly coming from the sun, is divided into three main categories: UVA, UVB, and UVC. As UVA and UVB reach the Earth, they can be harmful to living cells, causing various skin damage. Although UVA radiation has the least energy, it can reach deep skin layers, leading to the generation of free radicals and consequently long-term and indirect damage to DNA. Having higher energy, UVB radiation can cause direct damage to DNA and subsequently lead to skin cancer [1,2]. On the other hand, although UVC radiation has the highest energy, it is blocked by the atmospheric ozone layer; hence, it does not have a harmful impact on skin [3].

Sunscreens adopt two approaches to provide UV protection: (a) prevention of free radical generation (via UV filters) and (b) scavenging of free radicals (via antioxidants) [4]. UV filters act on the skin surface and tend to provide skin protection by absorbing, reflecting, and/or scattering destructive UV light [5]. However, even with the application of sunscreens of high sun protection factor (SPF), the epidermis is still exposed to low levels of UVR, leaning to the generation of UV-induced free radicals within the skin [6]. This explains the need to incorporate radical scavenging antioxidants into sunscreens’ formulations. Free radical neutralisation is achieved with a variety of antioxidants, often with a combination of both lipid- and water-soluble antioxidants to ensure complete protection. When added to cosmetic formulations, antioxidants interfere with the cascade of UV-induced free radical reactions, thus protecting the skin from oxidative stress.

An ideal sun protection formulation should provide a broad protection through the range of UVA to UVB (280–400 nm) radiation. It should also be designed to prevent UV filters from penetrating the epidermis given their potential toxicity, whilst enabling antioxidants to penetrate the viable layers of the epidermis and exert their effects [7]. Finally, the formulation should show good photo, chemical, and thermal stability. However, research over the past couple of decades has indicated that a multitude of sunscreens do not meet these requirements. In fact, UV filters, given their nature, can absorb UVR and consequently undergo photo-addition, photo-fragmentation, and rearrangement reactions [8]. These reactions often produce undesired free radical species, which can lead to the degradation of other ingredients within the cream formulation. Similarly, the affinity for antioxidants to free radical species translates to high susceptibility to oxidation and subsequent degradation. For instance, α-tocopherol (vitamin E), one of the most effective free-radical scavengers in modern skin care, exhibits poor UV stability and subsequently low activity times [4,9,10]. Additionally, lipophilic antioxidants have a tendency to aggregate, thus hindering their permeation of the skin and leading to poor bioavailability [11,12]. Finally, several UV filters have been detected in blood plasma following topical application. A recent report form the Food and Drug Administration (FDA) showed that six sunscreens UV filters were found to be present in blood plasma at concentrations that surpassed the FDA’s threshold [13].

Cyclodextrins (CDs) are cyclic oligosaccharides with the shape of a hollow truncated cone. CDs unique chemical structures allow them to entrap poorly soluble drugs, such as antioxidants, leading to significant enhancement of drug solubility and stability [14]. Therefore, certain formulation-related measures, such as using a CDs inclusion complex, could be undertaken to enhance antioxidants’ bioavailability and maintain the stability, efficiency, and safety of both antioxidants and UV filters throughout the sunscreen’s lifecycle and post application.

In this review, the impact of UVR radiation on skin is firstly introduced. Subsequently, sunscreens, UV filters, and natural antioxidants are reviewed considering the potential use of CDs to improve their solubility, stability, and biological efficiency. Finally, the inclusion complexes of UV filters and natural antioxidants with different CDs are critically assessed for their potential to enhance sunscreen’s therapeutic effect in preventing UVR-induced skin cancers.

## 2. Skin Structure and UV Radiation

Skin primarily consists of three layers: epidermis, dermis, and subcutaneous. The epidermis (50–100 µm) consists of non-viable epidermis and viable epidermis. Non-viable epidermis (10–15 µm), also known as the stratum corneum, is the uppermost skin layer that provides the primary barrier against the drugs permeation [15]. The viable portion of the epidermis (≈70 µm) is further subdivided into four layers; stratum lucidum (viable uppermost layer), stratum granulosum, stratum spinosum, and stratum basale [16]. The stratum basale is the closest layer to the dermis and contains various cell structures. These include keratinocytes, which produce the protective protein keratin, melanocytes, which synthesise melanin, Langerhans cells, where the main antigen-presenting cells (APC) are located, and Merkel cells that contain sensory neurons and stimulate sensations of touch and pain [17]. The majority of skin melanomas are present in the epidermis and can be divided into two main types: non-malignant melanoma and malignant melanoma. The most common non-melanoma skin cancers are basal cell carcinoma (BCC) (accounting for approximatly 75% of non-melanoma skin cancers) and squamous cell carcinoma (SCC), which in both cases affect the epidermal keratinocytes [18]. The dermis layer (3–5 mm) is the major constituent of the skin. The dermis is metabolically active and represents the main site of wound repairing. Therefore, it is essential for most drugs to reach this layer to exert therapeutic effects. Furthermore, the dermis contains various enzymes such as esterases, peptidases, and hydrolases, increasing the metabolic activity of the skin and reducing the skin bioavailability of drugs [19,20]. The subcutaneous tissue, known as the hypodermis, is the innermost skin layer that accommodates blood vessels and connective tissues [15].

UVR can be divided into three main spectrums: UVA (315–400 nm), UVB (280–315 nm), and UVC (200–280 nm) [3,21]. While the majority of UVR reaching the Earth is within the UVA spectrum, 5 to 10% comes from UVB. On the other hand, UVC is entirely blocked by the ozone layer; hence, it does not affect the biota. The radiation energy is inversely proportional to the wavelength; the longer the wavelength, the lower the energy. Conversely, the skin penetration depth is proportional to the wavelength. Therefore, UVA can penetrate deep layers of the epidermis, reaching the basal layer while UVB can only penetrate the epidermis, given its shorter wavelength.

**Figure 1:**
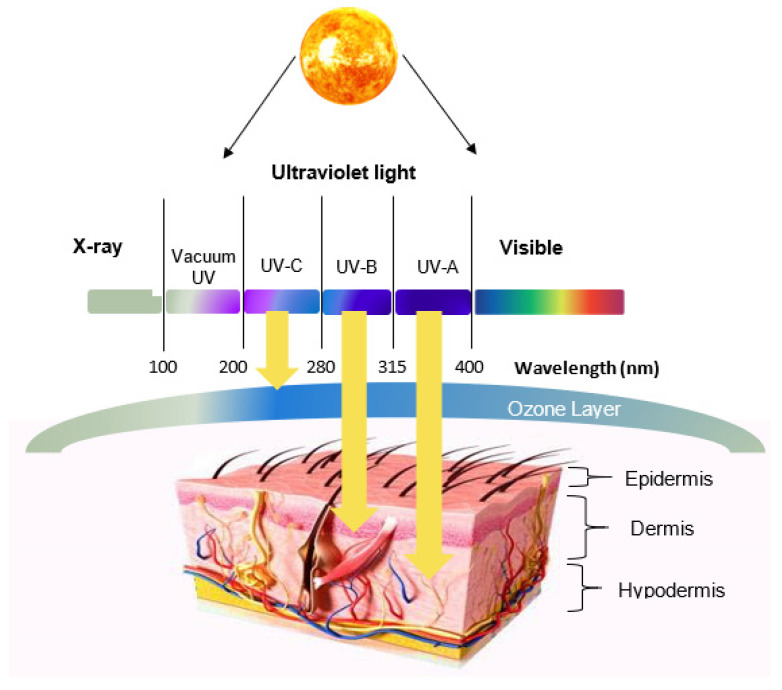
Cross-sectional view of human skin with the ultraviolet radiation (UVR) wavelength-dependent penetration of the skin.

Figure 1 shows a cross-sectional view of human skin along with the UVR wavelength-dependent penetration.

Upon sun exposure, the epidermal APCs are affected by the UVR immunosuppression. As a result, the number of these cells decreases, whereas the number of inflammatory cells increases. This contributes to the development of various diseases including skin cancer [22]. As UVA reaches the deep skin layers, it reacts with the skins endogenous photosensitisers. Once activated, photosensitisers initiate photosensitising reactions leading to the generation of reactive oxygen species (ROS) [23,24]. ROS are highly reactive species that induce oxidative cellular stress and alteration in cellular functionality. This leads to an indirect damage of DNA and consequently skin diseases, including skin cancer [25]. On the other hand, as UVB can only penetrate the non-viable upper layers of skin, it was thought that it does not affect DNA molecules, but it may only cause lighter skin damages such as suntan and sunburn [1]. However, later studies showed that due to its high energy, UVB can still cause molecular rearrangements and lead to DNA damage and cell cycle changes. Once the damage is done, mutations occur, and with their accumulation, genetic and molecular integrity of the DNA are disrupted, resulting in the first stages of skin cancer [23]. UVB can also activate cyclooxygenase-2 (COX-2) enzyme, increasing prostaglandins production and promoting inflammation [26]. Finally, both UVA and UVB were also found to stimulate the generation of superoxide anion radicals via the activation of the enzyme nicotinamide adenine dinucleotide phosphate (NADPH) oxidase and respiratory chain reactions [2].

The highest rates of skin cancer incidence in the globe are seen in Australia, where around 1% of the population develop BCC. Moreover, incidences of BCC and SCC in Australia are almost three times higher in populations closer to the equator with greater UVR [27]. It is well accepted that the risk of skin cancer is strongly correlated to UV exposure as a child/adolescent as well as prolonged exposure as an adult [28,29]. Malignant melanoma poses the greatest risk to human health of all the skin cancers [30]. As the fifth most common cancer in the UK, malignant melanoma accounts for around 80% of deaths by skin cancer [31]. Overexposure to solar UVR has been found as the cause of 86% of all melanoma cases.

## 3. Sunscreens

To prevent UVR skin damage, sunscreens were developed and introduced to the pharmaceutical and cosmetic market. Sunscreens are topical preparations, usually emulsions made of a two-phase system: oil in water (O/W) or water in oil (W/O). UV filters are the main active ingredient in sunscreen formulations [5].

### 3.1. UV Filters

Based on their mode of action, UV filters can be classified as (1) physical (inorganic) filters that reflect and scatter the light and (2) chemical UV filters that absorb the UVR (mainly UVB). The efficiency of UV filters is given by SPF value, indicating their ability to protect the skin against UVR; the higher the SPF, the better the sunburn protection [32]. While the maximum authorised concentrations of chemical UV filters in sunscreen formulations are usually reported at a range of 5–15% *w*/*w* of the formulation, physical UV filters could be added at higher concentrations (up to 25% *w*/*w*) [33,34]. Table 1 summarises some of the commonly reported physical and chemical UV filters used in sunscreen formulations.

To remain effective and pharmacologically inert, UV filters must only reside on the stratum corneum surface. The permeation of UV filters across skin not only compromises their photoprotection capacity but can also lead to severe toxic and allergic reactions [7]. Due to low permeability through skin layers, the potential for physical UV filters to cause hypersensitivity reactions is reduced [25]. However, physical UV filters tend to leave a white opaque layer on the skin surface, which is attributed to their scattering properties [2]. Therefore, the use of chemical filters is more commercially appealing [37]. To increase photoprotection, sunscreen formulations usually contain both chemical and physical UV filters. However, the combination could lead to photocatalytic and photolytic reactions, leading to the deterioration of their structure and efficacy [38]. The particle size of physical UV filters can affect the photostability of chemical UV filters. For example, the photodegradation of avobenzone was 12% higher when nanosized titanium dioxide particles (<25 nm) were used instead of micro-sized titanium dioxide (~0.6 μm). This was explained by the increased surface area that nanoparticles have, and therefore the greater amount of photocatalytically generated ROS [38].

### 3.2. Antioxidants

**Figure 2:**
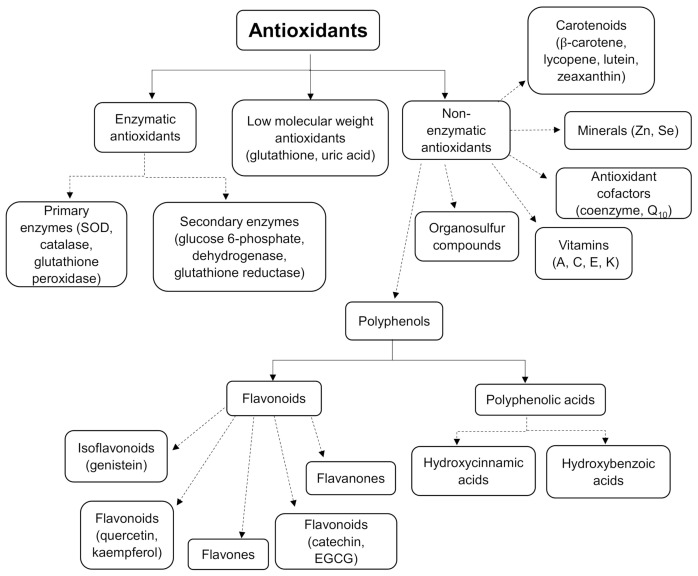
Classifications of antioxidants.

Antioxidants are a crucial group of compounds that protect the body from excessive free radical oxidation. Antioxidants can be categorised either as primary, secondary, or tertiary antioxidants or as enzymes, vitamins, flavanoids, carotenoids, phenolic acids, and polyphenols (Figure 2) [39]. Primary (or preventative) antioxidants work via the inbihibtion of radical initiation reactions to slow the rate of radical production. Secondary antioxidants, otherwise known as chain-breaking antioxidants, work by disrupting the propagation reactions, and tertiary antioxidants work by repairing the damage caused by free radical oxidation [40].

**Figure 3:**
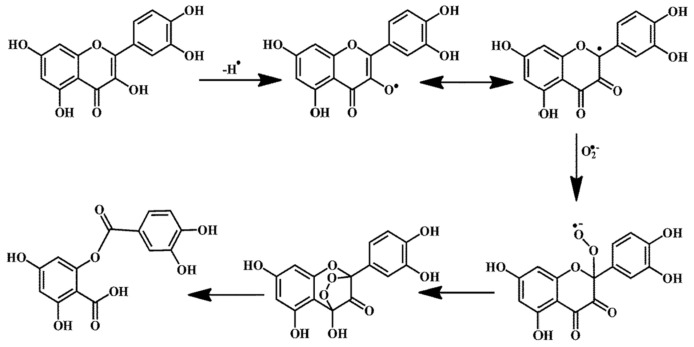
The radical scavenging mechanism of the superoxide anion by quercetin [44].

Antioxidants are added to sunscreen formulations to provide a second layer of defence, as UV filters alone cannot block 100% of UVR from reaching the skin (a product with SPF 50+ filters 98.3% of UVR). Unlike UV filters, antioxidants must penetrate deeper skin layers to reach their site of action. When applied to the skin, antioxidants’ main benefit resides in their ability to scavenge UV-induced ROS and free radicals, hence reducing DNA damage, skin ageing, and diseases [41,42]. There are many different mechanisms of action of antioxidants; two common examples of such are hydrogen atom transfer (HAT) and single electron transfer (SET) reactions. A single antioxidant can often work via more than one mechanism, depending on the site of action, the pH, and the radical being scavenged [28]. The influencing parameter in HAT reactions is the bond dissociation energy of the hydrogen-donating group of the antioxidant [29]. However, for SET reactions, it is the ionisation potential of the antioxidant that dictates its potency [27]. For this reason, some antioxidants are more suited to HAT than SET reactions and vice versa. Sunscreens often incorporate a selection of antioxidants with different radical scavenging properties to increase the formulation’s protection. Each antioxidant molecule reacts with one active free radical, transforming it into a non-radical species. This cycle continues until the ROS chain reaction is terminated [43]. Figure 3 shows the radical scavenging mechanism of quercetin (QCT), a flavanoid antioxidant, on the superoxide anion radical, O_2_^−^.

Although combining antioxidants with UV filters has a synergistic effect on the overall SPF value, it was reported that the antioxidant’s efficiency (i.e., radical protection factor) can be reduced upon the incorporation UV filters. Nonetheless, the SPF gained by this combination is still worth such a compromise [33]. Furthermore, antioxidants can help improve sunscreen formulation’s photostability and thus enhance their efficiency and safety [7]. In fact, the absorption of UV photons by a chemical UV filter, e.g., avobenzone, can lead to the formation of singlet oxygen free radicals, resulting in post-application degradation of the sunscreen product itself. Alfonso et al. reported that a combination of three antioxidants (ubiquinone, vitamins C, and E) stabilised UV filters, reduced the production of reactive species, and consequently increased the overall extent and duration of photoprotection [45].

**Figure 4:**
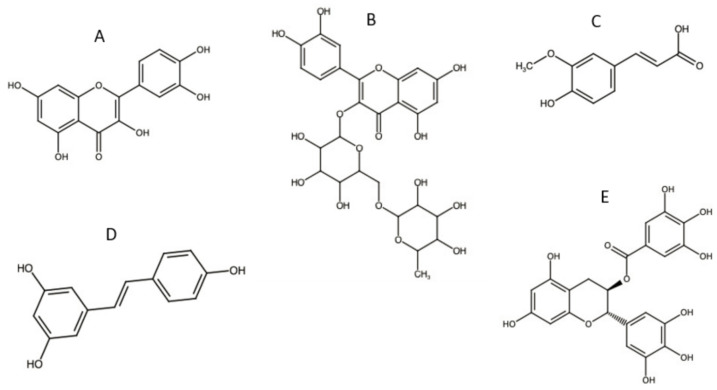
Chemical structures of commonly natural antioxidants complexed with cyclodextrins (CDs): (**A**) quercetin (QCT), (**B**) rutin, (**C**) ferulic acid, (**D**) trans- resveratrol, and (**E**) epigallocatechin Gallate.

Preferred by consumers, natural antioxidants stabilise sunscreen formulations and promote biocompatibility with reduced toxicity. Furthermore, natural antioxidants are more readily available and produce sunscreen products at a relatively low price compared to synthetic antioxidants [3]. Some commonly used natural antioxidants with their chemical structures are shown in Figure 4.

Flavonoids, such as QCT and rutin, are natural antioxidants that are frequently used in sunscreen formulations. These phenolic compounds have UV absorbing and anticancer properties [46,47]. As a result of the ROS scavenging capacity, QCT can prevent UVR-induced skin damage and inhibit the growth of tumour cells. Additionally, QCT has anti-inflammatory properties attributed to its capacity to inhibit the production of several inflammatory mediators such as the COX-2 enzyme [48]. Furthermore, it was reported that QCT can enhance the photostability of both UVA and UVB filters by inhibiting the activity of tyrosinase enzyme, hence decreasing photolabile activities on the skin [49].

Rutin is a non-toxic, non-oxidisable QCT derivative. Its chemical structure, including phenolic rings and free hydroxyl groups, gives it the ability to form intramolecular hydrogen bonds with free radicals (i.e., good antioxidant activity). Taira et al. analysed the scavenging abilities of rutin (extracted from Mallotus japonicus). It was found that rutin was able to inhibit tyrosinase activity and reduce the melanin content of murine melanoma cell lines by approximately 40% [49]. The extract showed no cytotoxicity, suggesting that it is safe for use in cosmetic and cosmeceutical preparations. Moreover, Corina et al. demonstrated that the antiproliferative activity of rutin was directly related to higher cellular uptake, leading to an increased apoptotic activity against cancer cells [50].

Ferulic acid (FA) is a phenolic compound with high antioxidant and radical scavenging activities. FA is also known for its anti-inflammatory effect due to its antiproliferative properties and the inhibition of the COX-2 enzyme [51]. Furthermore, FA helps to preserve the physiological cell integrity when exposed to air and UVR, thus preventing the formation of cancerous cells. Interestingly, FA also demonstrates UV-absorbing characteristics, promoting its use in sunscreen products [52].

Resveratrol (RES) is another phenolic compound known for its antioxidant and anticancer properties. RES can modulate the metabolism of lipids by protecting lipoproteins against damages induced by free radicals [53]. The trans-RES isomer has superior antioxidant, anti-inflammatory, and antitumoral properties [7]. It was found that trans-RES can inhibit lipid peroxidation more intensively than vitamin E or C [54]. This triple effect helps protect skin, fight diseases, and reduce edema, inflammation, and UVB-induced sunburn. Additionally, the anti-inflammatory properties of RES are correlated to its ability to inhibit photo-carcinogenesis.

Green tea polyphenols (GTP) are also used in topical applications, including sunscreens, for their antioxidant activity [55]. It was found that epigallocatechin gallate (EGCG), the main phenolic compound found in GTP, inhibited the synthesis of the COX-2 enzyme. This reduces the expression of hydrogen peroxide and consequently resists the generation of gene mutations and reduces the infiltration of leukocytes, which is a major source of ROS production [26].

The immense therapeutic potential of natural antioxidants is limited by their poor solubility and their formation of aggregates in water [56]. This results in poor bioavailability and low binding affinities to target receptors. Furthermore, natural antioxidants are also susceptible to degradation once exposed to oxidative factors such as light, heat, air, moisture, and UVR [11,12]. This explains the high doses of antioxidants in sunscreens in order to achieve the intended therapeutic effects. Therefore, the protection of antioxidants via formulation-related approaches is encouraged to enhance their stability and bioavailability.

The use of CD inclusion complexes in sunscreens is an emerging method for improving the incorporation of UV filters and antioxidants into formulations. Their ability to improve the solubility of lipophilic antioxidants and protect UV filters from photodegradation make it an appealing option. Solubility issues tend to be more prevalent with lipophilic antioxidants such as flavanoids, polyphenols, and carotenoids. For this reason, antioxidant–CD inclusion complexes with these categories of antioxidants offer greater potential value to the sunscreen industry than those complexes with already water-soluble antioxidants such as vitamin C and uric acid.

## 4. Cyclodextrins (CDs)

### 4.1. General Concept

**Figure 5:**
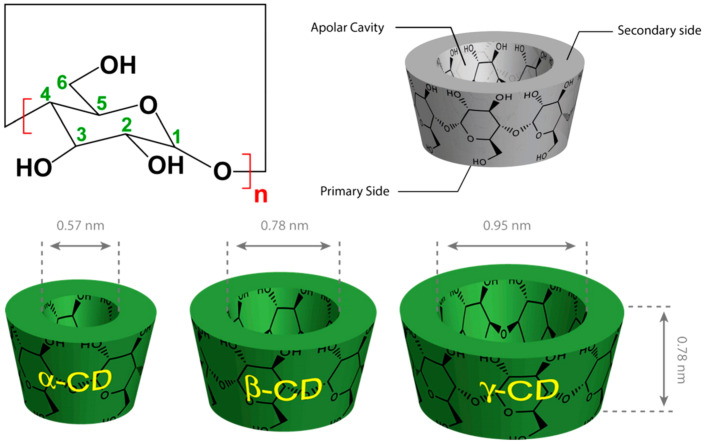
Types of cyclodextrins; α-CD (n = 6), β-CD (n = 7), and γ-CD (n = 8) with their geometric dimensions (bottom) [57].

CDs are cyclic oligosaccharides produced by the enzymatic degradation of starch, where the formed glucose residues link together by glycosidic bonds and form a macrocycle. Depending on the number of α-glucose units, CDs can be divided into three main types: α-CD, β-CD, and γ-CD, consisting of 6, 7, and 8 glucopyranose units linked by 1–4 bonds, respectively (Figure 5) [57,58]. CDs have a truncated-cone-shaped structure with hydrophilic surfaces and hydrophobic cavities. This unique structure allows them to entrap poorly soluble drug moieties, such as natural antioxidants and anticancer drugs, in their hydrophobic cavities to form inclusion complexes [59]. Several factors contribute to an effective formation of an inclusion complex. The guest molecule is efficiently maintained inside the cavity by noncovalent forces including hydrogen bonds, hydrophobic interactions, and van der Waals forces. The number of CDs glucose units determines the diameter and volume of its cavity (Figure 5) [60]. With eight glucose units, γ-CD have the largest cavity size, making it capable of accommodating relatively large antioxidants. However, larger cavities do not necessarily mean higher inclusion complex stability, as large cavities might allow an easy escape of guest molecules [61]. β-CD is the most used form in pharmacy, as its cavity size is the best fit for most drugs with molecular weight ranging between 200 and 800 Daltons [12]. However, due to its high lattice energy, which is attributed to the crystalline structure and intramolecular hydrogen bonding, β-CD solubility in water is relatively low [62]. Therefore, its chemical structure was modified to result in more amorphous, soluble, and potentially safer form of CDs [62,63]. For instance, the hydroxypropylated form (HP-β-CD) has higher solubility, reduced toxicity, and enhanced complexability compared with β-CD [64,65].

Different methods are used to prepare drug–CDs inclusion complexes, including co-precipitation, freeze drying, ball milling, and kneading [66,67]. The preparation method could affect the characteristics of the resulting inclusion complex [68]. Both freeze drying and co-precipitation methods yield higher entrapment efficiency; however, the freeze-drying method is costly and time consuming. In contrast, the kneading technique shows lower efficiency in entrapment capabilities [12]. Therefore, the co-precipitation method is more commonly used in preparing a stable inclusion complex of antioxidants [69].

CDs can act as a shield that protects the encapsulated molecules of antioxidants and UV filters, promoting their stability against external stimulus and oxidative stress [51,70]. The chemical stability of CDs is mainly attributed to the absence of reducing glycosidic units from their structures. The adjacent C-2 and C-3 hydroxyl groups of the glucopyranose units form hydrogen bonds that contribute further to the chemical stability of CDs’ structures and help promote solubility in water [57]. Furthermore, CDs are known to resist enzymatic hydrolysis due to burying all bridge oxygens inside the nonpolar ring center [71]. These bridges bind to the encapsulated antioxidants or UV filters, making them less available to interact with extracellular molecules [62]. Furthermore, due to the hydrophilic nature of their outer surface, CD-antioxidant inclusion complexes display enhanced solubility, hence enhanced bioavailability, and they can also be employed to modify the release of antioxidants from topically applied sunscreens [70]. Interestingly, following the application of CD-containing sunscreens, the CDs themselves can act as a protective film and UV filter adjuvant that absorbs UVR [72,73].

### 4.2. UV Filters/CDs Inclusion Complex

**Figure 6:**
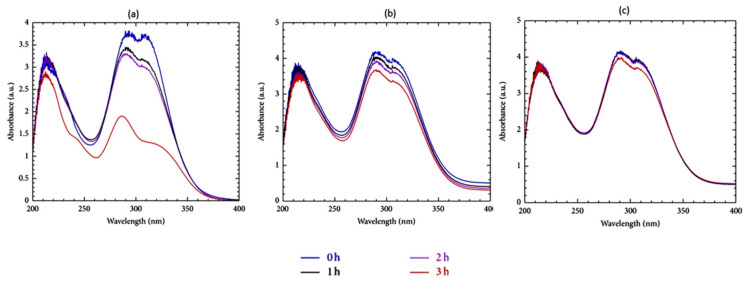
Change in UV absorption spectra of sunscreen sample containing a mixture of UV filters after different times of irradiation. (**a**) Free of β-CD; (**b**) 5 g β-CD; (**c**) 10 g β-CD [76].

CDs have been assessed for the protection of UV filters against photodegradation and oxidation and for their ability to restrict UV filters permeation into deep skin layers [74,75]. For example, β-CD was found to photostabilise different UV filters (oxybenzone, octocrylene, and ethylhexyl-methoxycinnamate). A significant photostability enhancement of UV filters was observed when β-CD was used compared to that in the absence of β-CD. It was found that in a cream sample containing no β-CD, there was a drop in the absorbance of λ_max_ = 287 and 309 nm, from ≈3.75 to ≈1.75 following 3 h of UV irradiation. However, with the inclusion of 5 g of β-CD, this drop in absorbance was dramatically reduced, and with 10 g of β-CD, the absorption profile remained almost constant after 3 h (Figure 6) [76].

4-Methylbenzylidene camphor (4-MBC, also known as enzacamene) is considered a relatively photostable organic UVB filter. However, a recent study showed that 4-MBC may decompose upon exposure to direct sunlight [77]. Scalia et al. reported that among different types of CDs, random methyl-β-CD (RM-β-CD) was found to have an optimal solubilising and photostabilising effect for 4-MBC. This was mainly attributed to CD’s ability to form a protective shield around 4-MBC and thus decrease the level of direct interaction with UVR [78].

**Figure 7:**
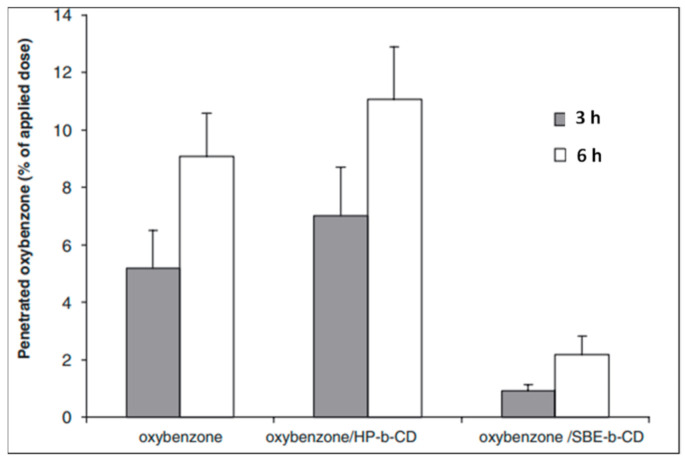
Penetration of oxybenzone across human epidermis to the receptor phase from solutions containing free or complexed sunscreen agent [81].

Chemical UV filters tend to penetrate the skin after topical application. This not only decreases their photoprotective capabilities but also leads to phototoxicity or photo-allergenicity. According to Scalia et al., the amount of avobenzone penetrating the stratum corneum was significantly reduced after encapsulation in HP-β-CD, with >70% of the applied dose remaining on the uppermost skin layer [79]. Furthermore, the permeation of UV filters was dependent on CDs concentrations; higher concentrations of HP-β-CD resulted in lower flux rates [80]. Likewise, the inclusion complex of oxybenzone in HP-β-CD and sulfobutylether-β-CD (SBE-β-CD) led to an increase in the aqueous solubility of oxybenzone (up to 1049-fold) whilst significantly limiting its percutaneous absorption (Figure 7) [81]. Furthermore, Shokri et al. reported that the absorption lag time and overall percutaneous flux of avobenzone, oxybenzone, and ensulizole were reduced by 4 to 15-fold after complexation with β-CD [82].

Due to its relatively low price and its absorption of both UVA and UVB radiation, UV filter benzophenone-3 is regularly used in sunscreen formulations. Its maximum permissible concentration in formulations is 6%, and its transdermal absorption can reach 2% after topical application [83]. A benzophenone-3–HP-β-CD complex was shown to enhance benzophenone-3 solubility and photostability and decrease its penetration through cell membranes. The permeability rates of benzophenone-3 were evaluated in vivo, and the benzophenone-3-HP-β-CD complex was found to have permeated less through the skin of healthy volunteers than the free form of benzophenone-3 [83].

The effect of employing CD-UV filters inclusion complexes on the overall SPF value of the formulation was investigated. While some studies reported negligible to minor increases in the formulation SPF when CD-UV filters inclusion complexes were used [84,85], other reported more significant effects. For instance, Srinivasan et al. measured the SPF values of two commercial sunscreens with and without β-CD complexes of different dinitro compounds (dinitrophenol, dinitroaniline, and dinitrobenzoic acid). The results showed that with the β-CD inclusion complexes, the SPF was increased by up to 19.6% [86]. Similarly, Felton et al. analysed the photoprotective effects of the HP-β-CD-oxybenzone inclusion complex. It was reported that a 5% HP-β-CD inclusion complex formulation provided sun protection equivalent to SPF 30 commercial sunscreen [87].

### 4.3. Natural Antioxidant/CDs Inclusion Complex

CDs provide antioxidant protection and enhance their solubility, stability, and hence their bioavailability and biological activity. Consequently, lower doses of antioxidants can be used, allowing better therapeutic index with reduced cost [11,37,88]. CDs are capable of entrapping large amounts of antioxidants. For instance, the loading capacity studies estimated an 80% inclusion of RES in the methyl-β-CD cavity [89]. Several factors contribute to a successful and strong formation of an antioxidant–CDs inclusion complex. These include the type of solvent used in the inclusion complex preparation, the pH, temperature, and mixing time. According to D’Aria et al., the optimised conditions that yielded the best solubility enhancement of the QCT–CDs inclusion complex were pH = 8.0 at 37 °C with a mixing time of 72 h [90]. In addition to temperature and reaction time, the mixing speed is also influential on the entrapment efficiency of antioxidants in CDs’ cavities [65]. Zhu et al. evaluated the influence of the mixing time, temperature, and molar ratio on the inclusion of chrysin (5,7-dihydroxyflavone) within the β-CD cavity. Temperature was reported to have the greatest effect on the inclusion rate, followed by the molar ratio and mixing time [91]. Table 2 summarises the main outcome of antioxidant–CDs complexation studies reviewed in this work.

#### 4.3.1. Antioxidant Solubility Enhancement

**Figure 8:**
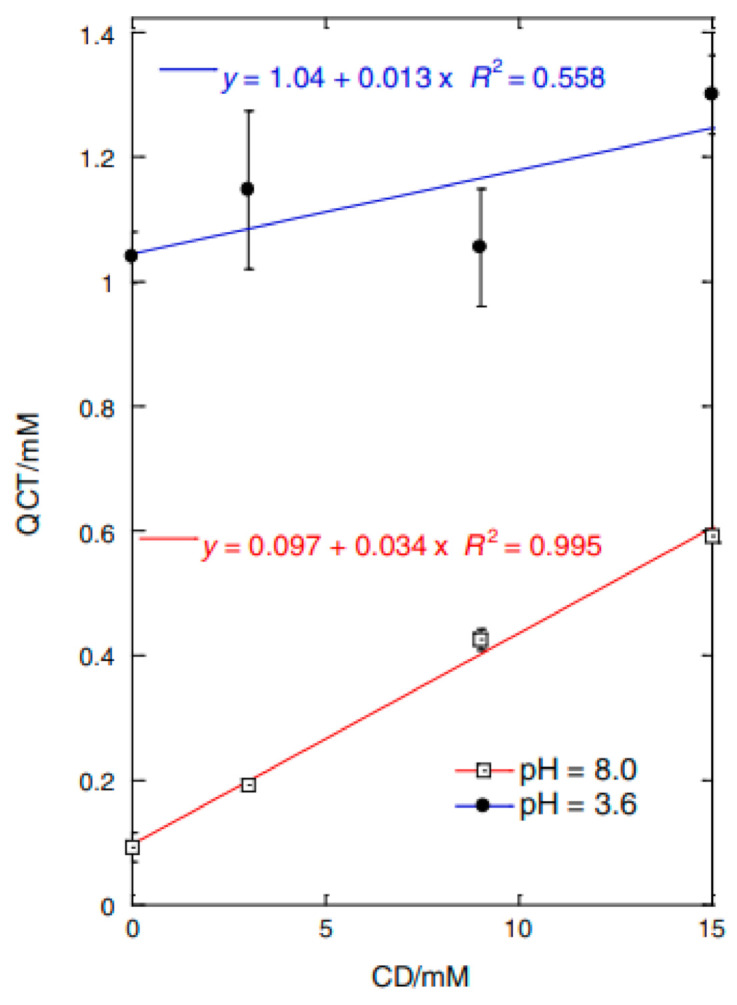
Phase solubility graph of QCT with HP-β-CD in phosphate (pH = 8.0) and citrate (pH = 3.6) buffers [90].

The phase solubility analysis is a useful method that is usually used to evaluate antioxidant–CDs solubility [100,106]. By plotting the molar concentration of solubilised antioxidant versus the molar concentration of CDs, the stability constant (Kc) can be determined using the intrinsic solubility (S_0_) and the slope of obtained diagram according to Equation (1) and as shown in Figure 8 [90].

(1) $$\mathrm{Kc}\;=\;\frac{\mathrm{slope}}{S_{0}\left(1-\mathrm{slope}\right)}$$

It is often assumed that a linear solubility diagram indicates a 1:1 stichometry. However, this method may not be sufficient to calculate the antioxidant to CDs stoichiometry or to confirm the presence of such a complex [107]. CDs and CD complexes tend to form aggregates that may be able to solubilise drugs via non-inclusion complexes. Furthermore, the fact that the Kc value determined by Equation (1) is dependent on the S_0_ value, which is usually inaccurately measured for poorly soluble compounds, makes this method less reliable. Therefore, Loftsson et al. introduced the complexation efficiency (CE) notion to determine the solubility of the guest drug as per Equation (2):

(2) $$\mathrm{CE}=S_{0}\times \mathrm{Kc}=\;\frac{\left[D∕\mathrm{CD}\right]}{\left[\mathrm{CD}\right]}=\;\frac{\mathrm{Slope}}{\left(1-\mathrm{slope}\right)}$$

where CE is calculated using the slope of the phase-solubility diagrams independently of both the intrinsic solubility of the drug, S_0_, and the intercept [108]. The stability constant and the stoichiometry of the formed complex can also be determined using other analytical techniques such electrospray ionisation mass spectroscopy [109] and nuclear magnetic resonance (NMR) [51].

Carlotti et al. reported that the inclusion complex of QCT in α- and β-CD helped improve its solubility and reduced photochemical reactivity [41]. Moreover, the water solubility of different flavonoids was enhanced by up to 100-fold after complexation with propanediamine-β-cyclodextrin (DP-β-CD) [98]. Similar solubility enhancement of RES was reported after complexation with HP-β-CD and RM-β-CD [68]. Furthermore, Me-β-CD, a modified version of β-CD, forms a more robust inclusion complex [110], allowing a 400 times enhancement in RES’s solubility [89].

**Figure 9:**
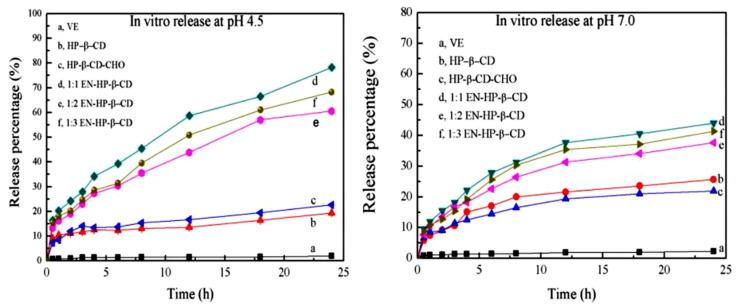
Cumulative release (%) of vitamin E, and its inclusion complexes with HP–β-CD and EN–HP–β-CD at pH 4.5. and pH 7.0 [103]. EN: ethylenediamine, HP: hydroxypropylated form.

Several studies proved that HP-β-CD enhanced the solubility of entrapped drug molecules significantly; however, its antioxidant entrapment efficiency is poor. Ethylenediamine (EN) derivative of HP-β-CD enhances its properties and further increases antioxidant entrapment and solubility [103]. For instance, while unmodified HP-β-CD was only able to entrap 17.72% of vitamin E, the entrapment efficiency increased to 51% using EN-HP-β-CD. This was mainly attributed to the crosslinked and porous structure of EN-HP-β-CD that was able to create stronger interactions with loaded vitamin E; hence, it enhanced further antioxidant solubility [103]. To produce the EN-HP-β-CD, HP-β-CD is oxidised using sodium periodate and crosslinked with ethylenediamine (EN) to form C=N bonds. In acidic conditions (e.g., the stratum corneum), C=N can be easily broken down, resulting in a pH-sensitive controlled release of entrapped drug as shown in Figure 9 [103].

#### 4.3.2. Antioxidant Stability Enhancement

CDs act as a shield that photo- and chemo-stabilises encapsulated antioxidants and protects them from enzymatic oxidation [41]. The photostability of rutin, rutin–β-CD, and rutin–HP–β-CD inclusion complexes was studied upon exposure to UVB irradiation. After 2 h of irradiation, the photodegradation percentages of rutin, rutin–β-CD, and rutin–HP–β-CD were 13.6%, 5.44%, and 2.52%, respectively, reflecting a significant improvement in rutin photostability after complexation with CDs [99]. Wang et al. evaluated the photostability of the FA–HP–β-CD inclusion complex. While free FA underwent 10% photodegradation in one hour of irradiation, it took more than eight hours of irradiation to reach 10% degradation after FA was complexed with HP–β-CD (i.e., the free form of FA experienced eight times faster degradation) [63]. Another successful example for using CD as a protection shield against UVB is RES–HP–β-CD inclusion complexes. Approximately 80–90% of trans-RES is converted to its less active isomer (cis-RES) after one hour of light exposure [111]. To overcome this limitation, Oliva et al. prepared RES–β-CD and RES–RM–β-CD inclusions complexes and reported a significant increase in RES solubility and photo and chemical stability after complexation [107]. In another study, RES–β-CD and RES–HP–β-CD inclusion complexes were prepared, and their potential to inhibit human cancer growth was investigated. While free RES had little inhibition on the viability of HeLa cells, both RES–β-CD and RES–HP–β-CD complexes exhibited high cytotoxicity on two human cancer cell lines (HeLa, Hep3B) with no significant effect on normal cells. Furthermore, the RES–HP–β-CD complex showed higher cytotoxicity than that of RES–β-CD [53]. Similarly, FA also undergoes structural transformation to its less active cis-isomer after UV irradiation. According to Monti et al., this phenomenon was not observed when FA was protected inside the α-CD cavity, indicating the resulting inclusion complex’s ability to provide a more stable active molecule [112].

#### 4.3.3. Antioxidants Activity Enhancement

**Figure 10:**
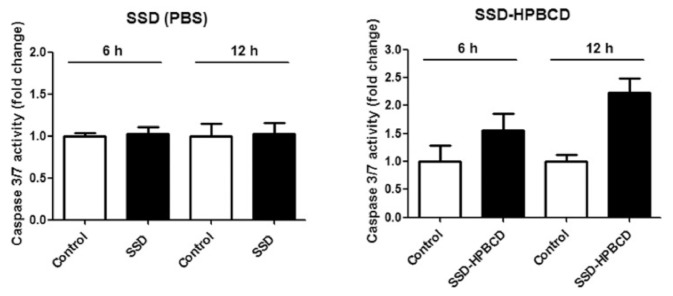
Effects of 2.5 μM pure saikosaponin-d (SSD) and SSD–HP–β-CD on HSC-1 cell apoptosis at 6 and 12 h [95].

The enhanced solubility and stability of antioxidants after complexation with CDs lead to enhanced antioxidant activity and biological efficiency. For instance, Zhu et al. reported that while the scavenging ability of pure chrysin was dramatically weakened after UV irradiation, that of the chrysin–β-CD complex was quite stable [91]. Similarly, the enhanced stability of the rutin–β-CD complex against degradative factors resulted in enhancing its scavenging capacity and bioavailability [99,104]. Hu et al. studied the antioxidant and anticancer effects (using the HSC-1 cell line) of saikosaponin-d (SSD), before and after complexation with HP–β-CD. It was found that 2.5 μM of SSD–HP–β-CD at a 1:5 molar ratio provided optimum cytotoxic effects, which were not seen using the pure form of SSD (Figure 10). This was attributed to the enhanced solubility of the SSD–HP–β-CD complex, which was reflected in its bioavailability and anti-cancer activities [95].

#### 4.3.4. Characterisation and Assessment

Different analytical techniques can be used to characterise antioxidant–CDs inclusion complexes. This includes differential scanning calorimetry (DSC), thermogravimetric analysis (TGA), infrared (IR) spectroscopy, nuclear magnetic resonance (NMR), X-ray diffraction (XRD), and UV stability.

Thermodynamic analysis is used to confirm the inclusion of antioxidants in CDs cavity and to evaluate the impact of temperature on antioxidants’ stability [113]. The differential scanning calorimetry (DSC) curve of CDs usually displays a broadened endothermic peak at 90–130 °C, which represents dehydration, followed by a decomposition endothermic peak at around 300 °C. On the other hand, the guest molecule DSC curve is often characterised by a well-defined sharp endothermic peak, indicating its melting point and hence its crystalline nature. Thermogravimetric analysis (TGA) on the other hand determines the weight of the sample with respect to change in temperature. Weight losses are usually observed at around 100 °C corresponding to CD dehydration and above 300 °C corresponding to the decomposition of the macrocycles. Any changes observed to the DSC and TGA curve after complexation, such as broadening, disappearing, or shifting peaks could indicate the loss of the crystallinity of the drug and the formation of an amorphous inclusion complex. Fir et al. used DSC and TGA thermograms (melting point positions, dehydration extent, and thermal decomposition) to confirm the formation of coenzyme Q10–CDs inclusion complexes and to assess their thermostability [93]. While uncomplexed coenzyme Q10 and CDs physical mixtures were stable up to 250 °C, coenzyme Q10–CDs inclusion complexes were stable up to 300 °C. Furthermore, the same study reported a significant enhancement in photostability after complexation [93]. The changes in enthalpy (ΔH^0^) and entropy (ΔS^0^) can also be used to confirm the spontaneous exothermic formation of chrysin–β-CD complex. Chakraborty et al. found that the complexation of chrysin with β-CD led to a decrease in entropy and a large negative enthalpy change, which can be explained by the increased hydrogen bonding and van der Waals forces between chrysin and CD, resulting in decreased rotational and translational freedom [102]. DSC analysis is also used to assess the physical state of CD inclusion complexes. Duarte et al. used DSC to characterise RES–Me–β-CD inclusion complexes. The DSC thermogram of unprocessed RES powder displayed a sharp melting peak at 270 °C, indicating its crystalline structure. However, this endothermic peak completely disappeared upon complexing with Me–β-CD, indicating the formation of an amorphous inclusion complex [89]. A similar observation was reported regarding the QCT–Me–β-CD complex, where the QCT melting endothermic peak at 300 °C disappeared after complexation [41]. In addition to DSC and TGA, both proton and carbon nuclear magnetic resonance spectroscopies (H-NMR and C-NMR respectively) are widely used to conclusively characterise CD inclusion complexes. Significant changes in resonance frequency after complexation can indicate the formation of the inclusion complex, the level of drug penetration in the CD cavity, and the moieties of the drug which are inside or outside the CD’s rim [51]. Al-Rawashdeh et al. used carbon-13 NMR for the characterisation of β-CD–sunscreen agents inclusion complexes [76]. The difference in 13C shifts between the free UV filters and the complexes helped confirm the structures. The authors postulated that these shifts can be ascribed to a change in shielding effects experienced once bound to CD. In addition, more specialised NMR techniques such as NOESY, ROESY, and DOSY NMR spectroscopy have also proven useful in the characterisation of CD inclusion complexes [114]. Since diffusion ordered NMR spectroscopy (DOSY) can distinguish between compounds of differing molecular weights within the complex mixtures, it demonstrated as particularly useful for identifying the CD inclusion complexes. Zhao et al. used this principle to distinguish between free isoflavones, free CD, and CD–isoflavones inclusion complexes [115].

An example of techniques used to assess the antioxidant activity is the colorimetric assay of 2,2-diphenyl-1-picrylhydrazyl (DPPH) solutions. DPPH is a stable free radical that forms a deep violet colour in organic solutions and undergoes discoloration once an antioxidant is introduced into the medium [61]. This method is widely used to assess the scavenging capacity of antioxidants, where the discoloration of DPPH solution quantitively determines the scavenging abilities of antioxidants [100]. DPPH reagent was used to evaluate the activity of an EGCG–γ-CD inclusion complex. It was found that the scavenged radicals were increased by 15% after EGCG complexation with γ-CDs [61]. Using the same method, Wei et al. assessed the scavenging ability of phloretin–HP–β-CD’s inclusion complex, where the scavenging abilities of free and complexed phloretin were found to be ≈48% and >60%, respectively [65].

Due to their low molecular weight (usually <500 Dalton) and lipophilic structure, natural antioxidants can permeate via the transcellular route through the epidermis layer. While essential for good permeability, the lipophilic nature of most natural antioxidants affects their dissolution rate and extent negatively. On the other hand, due to their high molecular weight (>972 Da) and hydrophilic nature (i.e., negative partition coefficient), CDs are unable to penetrate the lipophilic barrier of the skin. Instead, they adsorb at the stratum corneum, where the encapsulated molecules are then released [116,117]. In vitro assays are preliminary studies used to assess the antioxidants permeation through synthetic membranes simulating human skin. However, such assays are not considered adequate on their own to establish the activity of antioxidants. Therefore, ex vivo and in vivo assays, using porcine skin and volunteers, respectively, are used to evaluate the ability of the complex to diffuse antioxidants across skin layers [118]. QCT’s skin accumulation and percutaneous permeation were analysed ex vivo using Franz cells mounted with porcine skin. Pure QCT was mainly accumulated in porcine skin, with no noticeable percutaneous permeation. When a QCT–Me–β-CD complex was used, the amount of QCT retained in the skin was slightly lower; however, a substantial amount of QCT penetrated the skin from the complex after 24 h of application [41]. Spada et al. explored the in vitro, ex vivo, and in vivo skin permeation of milk thistle antioxidant. Both free milk thistle and complexed HP-β-CD were able to penetrate in vitro neither through the skin model nor ex vivo through skin layers. In contrast, the in vivo results demonstrated that the HP–β-CD complex effectively enhanced the skin penetration, with more than 80% of applied milk thistle penetrating through the upper skin layer [118]. The poor correlation between ex vivo and in vivo results was attributed to physical stress (i.e., high temperature) and the different treatment and viability of skin used ex vivo. These results emphasise the importance of incorporating in vivo studies to accurately assess CD’s ability to enhance drug permeation [118].

## 5. Conclusions

Chemical and physical UV filters are the main sunscreens’ active ingredients that protect skin against harmful UVR. Antioxidants are usually added to sunscreens’ formulation to counteract the harmful effect of UVR that was not successfully blocked by UV filters. However, antioxidants and UV filters are prone to degradation upon exposure to UVR and other environmental factors. This review shows that the inclusion complexes of antioxidants and UV filters in the CDs cavity represents a promising method to enhance sunscreens’ effectiveness and promote their stability. CDs provide a shielding cover that enhances both antioxidants’ and UV filters’ stability upon exposure to sun and oxygen. Furthermore, it was found that CDs could significantly enhance the aqueous solubility of poorly soluble antioxidants, boosting their biological activities, especially as anticancer. CDs can also control the release profile of encapsulated antioxidants and enhance their penetration through skin layers (requiring lower doses and hence minimalised associated risks) whilst simultaneously preventing the percutaneous permeation of UV filters.

## Figures and Tables

**Table 1:** Examples of UV filters used in sunscreen formulations [33,35,36].

Chemical UV Filters	Physical UV Filters
Benzophenones (UVA) Oxybenzone (UVA and UVB) Dioxybenzone (UVA and UVB) Avobenzone (UVA) Octocrylene (UVB) Homosalate (UVB) Ensulizole (UVB) Bemotrizinol (UVA and UVB) Octinoxate (UVB) Enzacamene (UVB)	Titanium dioxide Zinc oxide Iron oxide Kaolin Talc

**Table 2:** Summary of outcomes of antioxidant–CD inclusion complexes in reviewed studies.

Antioxidants	CDs	Inclusion Complex Preparation Method, Stoichiometry, and Main Outcome	Reference
Rutin	β-CD, HP–α-CD, HP–β-CD, HP–γ-CD	Inclusion complex prepared by mixing in solution with (1:1) stoichiometry with greater stability obtained using HP-β-CD and HP-γ-CD. Moderate protection of rutin against thermal and UVR degradation and significant enhancement of antioxidant capacity.	[56]
Astaxanthin	HP–β-CD	Inclusion complex obtained by freeze drying. Significant enhancement in antioxidant stability against light and oxygen, allowing controlled release.	[92]
Coenzyme Q10	β-CD, γ-CD	Inclusion complex prepared by co-precipitation method with (1:1) stoichiometry. Significant enhancement in antioxidant solubility and thermo- and photostability.	[93]
C60(OH)10	HP–β-CD	Inclusion complex/nanoparticles obtained by dry grinding using automated magnetic mortar. Significant enhancement in antioxidant scavenging ability, allowing better cell protection against oxidative stress. The IV administration of nanoparticles suppressed liver injury induced by oxidative stress.	[94]
Vitamin E	HP–β-CD	Inclusion complex prepared by mixing and electrospinning. Enhancement of vitamin E solubility, antioxidant activity, and light and shelf stability.	[43]
Phloretin	Me–β-CD, HP–β-CD	Inclusion complex prepared by freeze drying with (1:1) stoichiometry. Improved water solubility and antioxidant activities of phloretin.	[67]
Baicalein	HP–β-CD	Inclusion complex prepared by co-precipitation with compressed antisolvents method. In vitro and in vivo enhancement in Baicalein solubility, antioxidant activities, and bioavailability.	[69]
Lycopene	β-CD	Inclusion complex obtained by co-precipitation with (1:1) stoichiometry. Increased thermal and irradiant stabilities of lycopene.	[12]
Saikosaponin-d	HP–β-CD	Inclusion complex obtained by the freeze-drying method. Enhancement of water solubility allowing anticancer effects against cutaneous SCC cells.	[95]
Resveratrol	HP–β-CD	Inclusion complex obtained by mixing and electrospinning in the presence of polyvinylpyrrolidone. Resveratrol converted to amorphous form with intermolecular bonds with PVP and HP-β-CD. Good antioxidant activity and skin penetration and suppressed particulate matter-induced expression of inflammatory proteins.	[96]
Ferulic Acid	α-CD, β-CD, γ-CD, HP–β-CD, HP–γ-CD	Inclusion complex obtained by co-precipitation with (1:1) stoichiometry. α-CD was best in term of association constant, degree of photostability, and FA release.	[97]
QCT	Me–β-CD	Inclusion complex obtained by freeze drying. Enhanced QCT solubility and photostability, without significantly affecting the antioxidant activity or skin accumulation.	[41]
Taxifolin, QCT morin hydrate	DP–β-CD	Inclusion complex prepared by saturated aqueous solution method with (1:1) stoichiometry. Water solubility increased by 70 to 102 times with improved antioxidant activity.	[98]
Rutin	β-CD, HP–β-CD	Inclusion complex obtained by co-precipitation with (1:1) stoichiometry. Enhanced antioxidant activity, solubility, and photostability.	[99]
Curcumin	β-CD crosslinked polymer	Inclusion complex obtained by pressure distillation and oven drying resulting in (1:1) stoichiometry. Improved physicochemical characteristics, novel AO activity, with higher antiproliferative activity on A375 cell, and A375 cell apoptosis.	[100]
Ferulic Acid	γ-CD	Inclusion complex obtained by co-precipitation. Good encapsulation/release of ferulic acid for pharmaceutical application and biological activity.	[101]
Ferulic Acid	α-CD	Inclusion complex obtained by co-precipitation with (1:1) stoichiometry. Slower release, with improved photostability and bioavailability.	[51]
Ferulic Acid	HP–β-CD	Inclusion complex obtained by freeze drying and improved water solubility and bioactivity.	[64]
Chrysin	β-CD	(1:1) stoichiometry. The inclusion complex formed via A-ring of chrysin. Increased solubility and antioxidant potential.	[102]
Epigallocatechin Gallate	γ-CD	(1:1) stoichiometry. Slight enhancement in antioxidant activity.	[61]
Different flavanols	HP–β-CD	Increased antioxidant activity was attributed to CD capacity of protecting flavanols against rapid oxidation by free radicals.	[60]
Vitamin E	HP–β-CD EN–HP–β-CD	Inclusion complex prepared by mixing in solution with stoichiometry of (1:1). EN-HP-β-CD improved vitamin E solubility by 25 times. Higher release in pH 4.5 (pH-sensitive properties).	[103]
Ferulic acid 012	γ-CD	Inclusion complex obtained by co-grinding using a three-dimensional ball mill with a stoichiometry of (1:1). Five-fold enhancement in solubility.	[52]
Rutin	β-CD HP–β-CD	Inclusion complex obtained by co-kneading method. Improved antioxidant activity with antiproliferative and pro-apoptotic activities against B164A5 cells.	[50]
Rutin	β-CD	Inclusion complex obtained by co-grinding method with (1:1) stoichiometry. Greater stability and solubility, but antibacterial effect was slightly decreased. Prolonged released was observed with twice as much rutin skin permeation.	[104]
QCT	β-CD, HP–β-CD, SBE–β-CD	(1:1) stoichiometry. Inclusion ability of investigated CDs was in the following order: SBE-βCD > HP-βCD > βCD. All obtained complexes showed enhanced scavenging capability.	[17]
Phloretin	HP–β-CD	Inclusion complex prepared by simple co-evaporation method with (1:1) stoichiometry. The aromatic ring of phloretin was included into the HP-β-CD cavity from the narrow side. Enhanced solubility (by 5808 times) and stability, with preserved radical-scavenging capacity.	[65]
Chrysin	β-CD	Inclusion complex obtained by mixing in solution with (1:3) stoichiometry and inclusion rate of 90%. Increased solubility, antioxidant, antimicrobial, and antitumour activities.	[91]
Resveratrol	Me–β-CD	(1:1) stoichiometry. Enhanced solubility (400-fold) and good antibacterial and antioxidant activities with no haemolytic effect.	[89]
Garlic Oil	β-CD	Inclusion complex obtained by co-precipitation with (1:1) stoichiometry. Improved solubility with controlled release profile.	[105]
